# Stromal characteristics may hold the key to mammographic density: the evidence to date

**DOI:** 10.18632/oncotarget.6912

**Published:** 2016-01-13

**Authors:** Alastair J. Ironside, J. Louise Jones

**Affiliations:** ^1^ Centre for Tumour Biology, Barts Cancer Institute, Queen Mary University of London, London, UK

**Keywords:** mammographic density, breast cancer, stroma, extracellular matrix, collagen

## Abstract

There is strong epidemiological data indicating a role for increased mammographic density (MD) in predisposing to breast cancer, however, the biological mechanisms underlying this phenomenon are less well understood. Recently, studies of human breast tissues have started to characterise the features of mammographically dense breasts, and a number of *in-vitro* and *in-vivo* studies have explored the potential mechanisms through which dense breast tissue may exert this tumourigenic risk. This article aims to review both the pathological and biological evidence implicating a key role for the breast stromal compartment in MD, how this may be modified and the clinical significance of these findings. The epidemiological context will be briefly discussed but will not be covered in detail.

## EPIDEMIOLOGY OF MAMMOGRAPHIC DENSITY

Mammographic density (MD) refers to the proportion of a mammogram occupied by radiologically dense fibroglandular tissue and is a major independent risk factor for breast cancer. A comprehensive meta-analysis of 14,000 cases observed that women with > 75% breast density had 4-6 times the risk of developing breast cancer compared to women with breasts of the lowest 25% density [1]. The relative risk imparted by high MD is greater than family history or menstrual and reproductive risk factors, only age and BRCA mutation status are associated with a higher relative risk [2]. However, given the high frequency of breast density in the general population, the attributable risk is substantial and it is estimated that approximately one third of breast cancers could be explained by density in more than 50% of the breast [3]. High MD is also associated with increased local recurrence and risk of second primary breast cancer [4, 5].

MD appears to be highly heritable; studies of twins have attributed up to 65% of the variation in MD to genetic factors [6, 7] with the remaining 35% being modifiable.

The majority of studies investigating the genetic contribution to MD have focused on investigating the single nucleotide polymorphisms (SNPs) associated with increased breast cancer risk and their relationship with MD. So far SNPs in the region of ESR1, CCDC170, EBF1, LSP1, MIR1972-2:FTO, RAD51L1, ZNF365, MKL1, TNRC9/TOX 3, NTN4, NEK10, TAB2 and loci at 2p24.1 and 12q24 [8–14] have all been identified as having a significant association with MD. In addition, a recent meta-analysis of 10,727 women found that 18% of breast cancer susceptibility variants were associated with at least one MD measure [14]. Despite this, these SNPS are thought to account for only a small percentage of the variance in breast density with the remainder attributed to genes that are currently unknown [9]. Studies investigating more established genetic mutations known to confer a strong risk for breast cancer have focused on BRCA1 and BRCA2, and have demonstrated no significant association with MD [15, 16]. To date, no studies have investigated other established genetic risk factors such as phosphatase and tensin homolog (PTEN), tumour protein 53 (TP53), E-Cadherin (CDH1) or serine/threonine kinase 11 (STK11) for association with MD. Therefore much of the remaining genetic contribution to MD remains to be elucidated.

## MODIFIERS OF MAMMOGRAPHIC DENSITY

A key feature of MD compared to other established risk factors for breast cancer is that it is dynamic and modifiable. This modifiability offers significant therapeutic potential in the form of cancer prevention strategies targeted to reduce breast density. MD typically decreases with age, whereas breast cancer incidence conversely increases with advancing age. The Pike model attempts to resolve this apparent paradox by suggesting that cumulative lifetime exposure of the breast to dense tissue and associated growth factors and hormones, also referred to as ‘breast tissue ageing’, confers the age-related risk of developing breast cancer [17].

This is reflected in the apparent hormonally responsive nature of MD; women with known risk factors for breast cancer related to prolonged oestrogen exposure, such as late first pregnancy and early menarche, show a higher degree of MD. In addition, the use of combined hormone replacement therapy also increases MD [18, 19]. Conversely parous women have a lower degree of MD (approximately a 10% reduction per live birth [20]) and there is a significant reduction in MD following the menopause [21, 22]. An important confounding factor is that MD is also negatively associated with body mass index (BMI), an independent risk factor for breast cancer [20, 23]. High BMI reduces percentage density by increasing the non-dense portion of the breast and has also been associated with reduced absolute density in some studies [24, 25], however this is not a consistent finding [26, 27]. Failure to correct for BMI may lead to a significant underestimation of risk [23].

Given that MD appears to be hormonally responsive, one might expect high MD to predispose to the development of oestrogen receptor (ER) positive tumours, however to date studies have reported inconsistent findings. McCormack and dos Santos Silva have recently performed a systematic meta-analysis incorporating over 24,000 cases, finding a similar magnitude of association with ER+ and ER- tumours and no difference in the association with human epidermal growth factor receptor 2 (HER2) status [28].

Tamoxifen is a selective oestrogen receptor modulator, demonstrated in the IBIS-1 study to reduce MD and breast cancer risk [29]. A further nested case control study within the IBIS 1 cohort revealed that, in those women taking tamoxifen, those who showed a > 10% reduction in MD had a 63% lower risk of developing breast cancer [30]. Furthermore, those women who showed a poor response to tamoxifen treatment (< 10% reduction in MD) showed no associated risk reduction. Thus the protective effect of tamoxifen appears associated with a reduction in MD. This has been further highlighted in a recent retrospective study of breast cancer patients who received adjuvant tamoxifen for 15 years [31]. A 20% relative reduction in MD was reported for women who took tamoxifen and this reduction was associated with a 50% risk reduction in breast cancer mortality [31].

Presently the mechanisms conferring susceptibility to the protective effect of tamoxifen treatment remain unknown, however, an association of cytochrome P450 2D6 (CYP2D6) metabolizer status with MD change in response to tamoxifen has recently been reported [32].

In an animal model of breast cancer, tamoxifen treatment initiated remodelling of the mammary stroma to a tumour inhibitory phenotype with lower levels of fibronectin and reduced extra-cellular matrix (ECM) turnover [33]. Furthermore a recent study utilising a xenograft model of high MD human tissue maintained in murine biochambers showed that tamoxifen treatment resulted in a reduction in radiographic tissue density characterised histopathologically by a decrease in stromal tissue and increase in adipose tissue [34]. The evidence from these studies suggest that tamoxifen may modulate density and breast cancer risk by modifying the stromal microenvironment of the breast.

## PATHOLOGICAL CORRELATE OF MAMMOGRAPHIC DENSITY

MD reflects variations in the tissue composition of the breast and is positively associated with collagen, epithelial and stromal cells and negatively associated with fat [35]. Stroma is the major tissue component of the breast and is composed of stromal cells (fibroblasts, endothelial cells, immune cells and adipocytes) and ECM proteins, the most abundant of which is collagen I. A number of studies have indicated that mammographic density corresponds more to alterations in stromal composition rather than epithelial changes [36–39].

A comprehensive study by Li and colleagues examined histological features of breast tissue obtained at forensic autopsy [35]. They found that the area of stromal collagen was most strongly associated with percentage density and accounted for 29% of the variation in percentage density whereas nuclear area and glandular area accounted for between 4 and 7% of the variation [35]. Similarly, Pang et al have recently reported a significant association of increasing MD with increased proportion of fibrous stroma [40]. In addition, Huo and colleagues have reported more organised stromal collagen present in high MD breast tissue compared to low MD tissue [41]. Thus there are potentially both quantitative and qualitative aspects to the stromal differences in high MD, both of which need to be considered when trying to dissect underlying biological mechanisms.

It has been hypothesised that MD may represent the influence of local oestrogen production on the breast [42]. However, systemic levels of oestrogen have so far only shown an inverse or no association with MD [43]. Serum levels of prolactin and insulin-like growth factor 1 (IGF-1) have been associated with MD in a number of studies [44, 45]. In addition, dense breast tissue has been associated with higher levels of tissue inhibitor of metalloproteases 3, higher immunohistochemical expression of IGF-1 [46] and with stromal proteoglycans lumican and decorin [36]. Furthermore, genetic polymorphisms in several components of the insulin-like growth factor (IGF) pathway also show an association with increased MD [47].

Despite the association of stromal content and high MD breast tissue, there is no standardised approach to measuring MD in histological sections. This, combined with the fact that MD is often heterogeneous in nature throughout the breast, poses a significant challenge to the planning of translational studies using human tissue.

## BIOLOGICAL MECHANISMS CONTRIBUTING TO THE DENSITY-ASSOCIATED RISK

Whilst there is a strong clinical correlation, MD has not yet been causally linked to tumour formation and there have been a limited number of studies investigating the biological pathways mediating MD. Given that the stroma forms the major constituent component of dense breast tissue, the pathways contributing to the density-associated breast cancer risk are likely to involve the stromal cells, ECM proteins, and their interaction with the epithelial component.

The stromal microenvironment is known to have an important role in the progression of established invasive breast cancer [48], acting *via* multiple diverse mechanisms including the influence of growth factors secreted by cancer associated fibroblasts and remodelling of the ECM [48, 49]. In addition, ECM gene expression levels are also associated with breast cancer prognosis [50], response to neo-adjuvant chemotherapy [51] and endocrine therapy [52]. Similar stromal mechanisms may also have a role in promoting tumourigenesis in breast tissue of high MD; the evidence for this is reviewed below.

### Paracrine factors

It has been hypothesised that in areas of high MD, abundant stromal fibroblasts may aberrantly secrete growth factors and hormones/cytokines that stimulate epithelial cell proliferation [46, 53]. One study has suggested that local oestrogen production in the breast may be responsible for determining density [42]. Local oestrogen is synthesized from androgens in the breast by aromatase enzyme activity. Studies from Vachon et al and Huo et al have both reported increased aromatase immunoreactivity in the stromal cells of mammographically dense regions of the breast compared to non-dense regions [41, 42]. Increased stromal aromatase activity could result in sufficient local oestrogen production to stimulate epithelial cell proliferation and drive tumourigenesis. These findings are supported by *in-vitro* work from another group who have observed that cell density, shape and ECM proteins are capable of inducing stromal aromatase expression [54], thus providing a potential mechanistic link. This study also highlighted a role for IκB-kinase-β (IKKβ) as a key messenger in mediating this response. Figure 1a summarises the potential contribution of paracrine and mechanical factors to the induction of stromal cell aromatase activity.

**Figure 1a:**
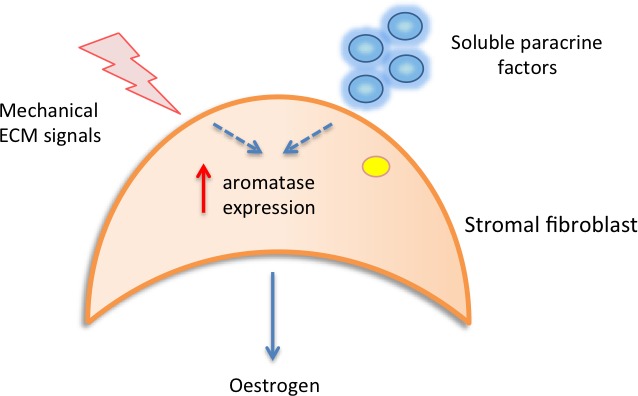
Local paracrine and mechanical signals may contribute to increased stromal aromatase expression, resulting in increased local oestrogen production and epithelial cell proliferation

### Collagen density, force and ECM stiffness

It has been suggested that increased collagen production by stromal fibroblasts contributes to a stiffer ECM. The inability of normal breast epithelium to contract stiff matrix causes a tensional imbalance, reflected by altered signalling pathways that have the potential to induce epithelial cell proliferation [55].

Patricia Keely and colleagues developed a bi-transgenic mouse to model high breast density, utilising the Col1a1^tmJae^ transgene which reduces collagen proteolysis, and examined the propensity for tumour formation with increased mammary collagen. They observed that higher stromal collagen density in mouse mammary tissue resulted in a threefold increase in tumour number and that the tumours which developed displayed a more invasive phenotype with greater local invasion and metastasis. The authors propose a functional link between increased stromal collagen density and breast cancer initiation and progression [56].

Two possible mechanisms are suggested by which increased collagen density might promote tumour development. The first is a direct effect of increased matrix stiffness, resulting in a higher mechanical force and resistance to contractility on the epithelial cells. These forces might alter focal adhesion and Rho GTPase (Rho) signalling, resulting in increased proliferation [56]. The second proposed mechanism is more indirect and suggests that collagen density modulates the behaviour of mammary fibroblasts, resulting in altered secretion of soluble growth factors and chemokines such as transforming growth factor beta (TGF-β), epidermal growth factor (EGF) and IGF, which in turn influence epithelial cell behaviour [56], as summarised in Figure 1b.

**Figure 1b:**
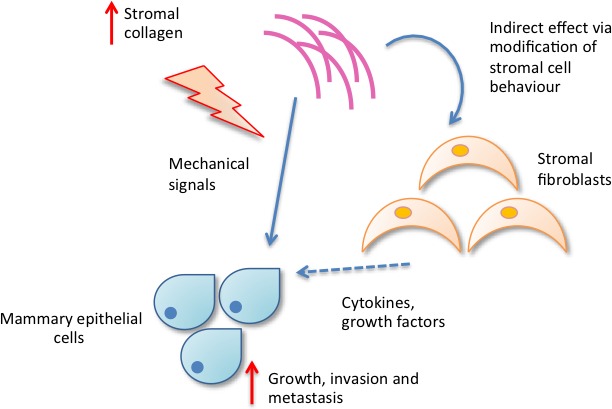
Increased collagen density may influence mammary epithelial cell behaviour directly *via* mechanistic signals or indirectly *via* modifying stromal cell behaviour

Further work by this group examined whether collagen density alone, in the absence of fibroblasts, could alter mammary epithelial cell behaviour in 3D culture models. Supporting this hypothesis, greater collagen density alone increased matrix stiffness and promoted epithelial cell proliferation and invasion [57]. They showed that mammary epithelial cells formed clustered 3D matrix-adhesions containing activated focal adhesion kinase (FAK) in response to matrices of high stiffness. FAK, Rho and extracellular signal-related kinase (ERK) were all found to be necessary for successful mechanotransduction of mammary epithelial cells, and inhibition of ERK or the Rho/Rho-associated protein kinase (ROCK) pathway reverted the invasive phenotype promoted by high density matrices [57].

The authors propose that continued mechanical stimulation from a dense stromal matrix results in sustained activation of FAK-Rho signalling which, in turn, up-regulates other pathways such as Ras- Mitogen activated protein kinase (MAPK). ERK acts as a crucial bottleneck, regulating the response and inducing transcription of proliferation associated genes.

Soon et al examined the effect of high density collagen matrices on mammary epithelial cell behaviour. They found that high density matrices up-regulated the expression and activity of Rho-associated coiled-coil-containing protein kinase 1 (ROCK 1) *via* inhibition of notch homolog 1 (NOTCH1) [58]. ROCK- 1 is proposed to have a key role in cell contractility and facilitating epithelial cell migration in conjunction with matrix-metalloproteinase (MMP) proteolytic activity, thus providing an alternative mechanism for density modulation of cell behaviour.

As well as collagen density, the significance of collagen fibre alignment within the ECM in facilitating mammary tumour cell invasion has also been investigated by the Keely group [59]. Collagen changes at the tumour/stroma boundary termed ‘Tumour associated collagen signatures’ (TACS) have been classified and used as markers of malignant progression. TACS-3, where the collagen fibers are orientated perpendicular to the tumour boundary, has been shown to correspond to sites of focal invasion in mouse models [56, 59] and in an analysis of human tissue samples is also associated with poor disease-specific and disease-free survival [60]. The precise biological mechanisms mediating alignment of the ECM remain unclear though it has been suggested that syndecan signalling, cyclooxygenase-2 (COX-2) signalling, stromal immune cells and other glycoproteins such as neutrophil gelatinase-associated lipocalin (NGAL) may play a role [55]. These data emphasise the importance of assessing both qualitative and quantitative elements of the stroma when considering the impact of mammographic density.

### Collagen cross-linking, integrin signalling and ECM stiffness

Weaver and colleagues have investigated how collagen cross-linking mediated by lysyl oxidase (LOX), a family of ECM cross-linking enzymes, can influence mammary epithelial cell behaviour. They used the MMTV-Neu mouse model of breast cancer to show that fibroblasts overexpressing LOX form a stiffer mammary fat pad and promote the growth and invasion of premalignant mammary epithelial cells. Similarly, inhibition of LOX resulted in a less stiff matrix and reduction in tumourigenesis [61]. LOX-mediated collagen cross-linking resulted in a stiff matrix characterised by increased focal adhesions, integrin clustering and subsequent increase in integrin signalling, increased FAK activity and enhanced phosphotinositide 3-kinase (PI3-K) signalling. PI3-K signalling has been suggested to promote invasion of mammary epithelial cells *in-vitro* and tumourigenesis *in-vivo* [62]. Thus it is proposed that LOX may have a pivotal role in modulating breast tumour progression by stiffening the ECM and initiating integrin mediated mechanotransduction of mammary epithelial cells. In keeping with these findings, LOX expression is also elevated in many cancers [63], has been associated with prognosis [64] and is proposed to have a key role in facilitating tumour metastasis [65].

**Figure 2:**
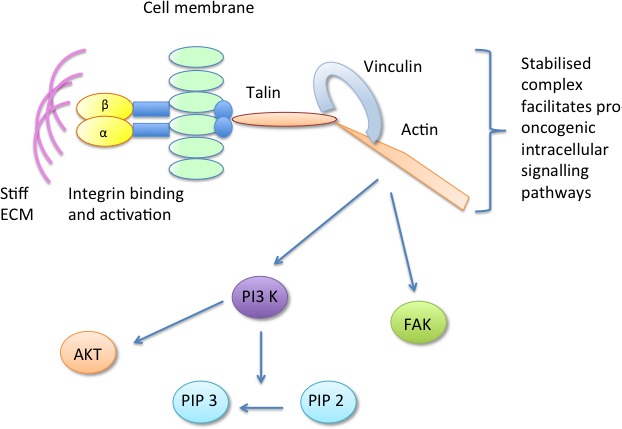
The focal adhesion component vinculin is activated in response to stiff ECM, forming a stable talin-vinculin-actin complex which promotes pro-oncogenic signalling pathways

More recent work by the same group, focusing on the molecular determinants of mammary epithelial cell mechanotransduction, has highlighted a crucial role for the focal adhesion component vinculin in translating the mechanical cues from a stiff ECM into tumour promoting intracellular signalling pathways [66]. It is suggested that a stiff ECM drives integrin binding and activation, forming focal adhesions. At the focal adhesion, vinculin is activated and binds talin and actin forming a stable talin-vinculin-actin complex. The stabilisation of this complex facilitates PI3-K conversion of phosphatidylinositol 4,5-bisphosphate (PIP2) to phosphatidylinositol (3,4,5)-trisphosphate (PIP3), phosphorylation of FAK and protein kinase B (AKT) and upregulation of pro-tumourigenic signalling pathways [66]. Thus, common mechanisms for mammographic density signalling, centering around focal adhesions and the cytoskeleton, are becoming apparent, as summarised in Figure 2.

**Figure 3:**
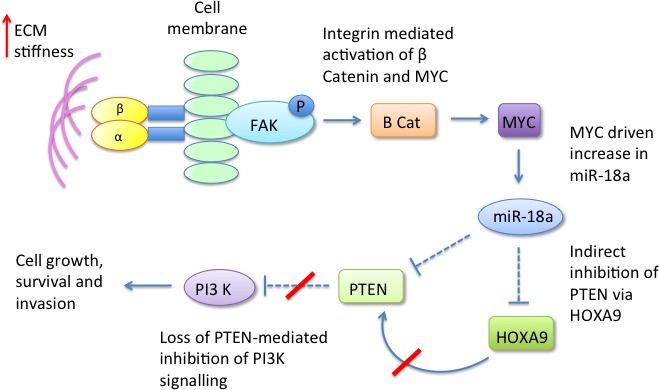
Dense ECM induces expression of micro RNA 18a *via* activation of MYC and beta catenin miR-18a inhibits PTEN both directly and indirectly *via* HOXA9, resulting in upregulation of PI3-K signalling.

The Weaver group have also recently reported that changes in ECM stiffness can modulate micro RNA expression [67]. They demonstrated that increased matrix stiffness in both human and mouse mammary tissue induces expression of micro RNA-18a (miR-18a) *via* integrin-dependent activation of β-catenin and MYC. miR-18a subsequently interacts and inhibits expression of the tumour suppressor gene PTEN, which itself is a negative regulator of PI3-K activity - previously demonstrated as a key pathway promoting mammary epithelial cell malignant progression [61]. miR-18a was also noted to indirectly inhibit PTEN expression *via* decreasing levels of homeobox A9 (HOXA9) (Figure 3). In this study miR-18a levels were able to distinguish luminal A from luminal B tumours and high miR-18a expression was predictive of poor outcome in tissue biopsies of patients with luminal breast cancers, suggesting that this pathway could be utilised clinically. Whether this could be used to monitor patient response to chemopreventive measures remains to be determined.

### Reduced expression of CD36

One of the key features of high mammographic density is the change in balance of adipose tissue to fibrous stroma, and it might be anticipated that factors determining adipose differentiation may be altered. A recent study from Tlsty and colleagues has suggested a critical role for the transmembrane receptor CD36 in mediating MD by controlling two key density-determining factors: stromal adipocyte content and ECM accumulation [68]. Gene expression profiling was used to determine differentially expressed genes in fibroblasts from high and low density disease-free breast tissue and cancer associated fibroblasts. CD36 expression was repressed in a range of stromal cell types (fibroblasts, adipocytes, endothelial cells) in both tissues of high MD and in tumour stroma. Loss of stromal CD36 expression *in-vitro* and *in-vivo* resulted in less fat accumulation and greater ECM accumulation, a phenotype shared by tissues of high MD and desmoplastic tumour stroma [68]. They have suggested that reduced CD36 expression across multiple stromal cell types results in a complex, coordinated set of signalling pathways which increases the risk of tumour development *via* a variety of mechanisms including adipocyte differentiation, angiogenesis, cell-ECM interaction and immune signalling. In addition, clinically more aggressive tumours were associated with a greater degree of CD36 repression [68]. These findings highlight the potential importance of CD36 as a targetable stromal biomarker of MD associated risk in the cancer prevention setting.

Further work by this group has demonstrated that epithelial cells in high MD tissue show more DNA damage signalling, shorter telomeres and increased activin A secretion compared to low density tissue [69]. In addition epithelial expression of activin A and telomere dysfunction were capable of reducing CD36 expression in the surrounding stromal fibroblasts, suggesting a potential pathway by which high MD tissue might arise. Whether the initiating DNA damage event occurs in the epithelial or stromal compartment of the breast remains to be elucidated, but these studies highlight the dynamic interaction between stromal and epithelial compartments.

### JNK1 stress signalling and myofibroblast phenotype

Further interrogating gene expression array data from low- and high-density fibroblasts, Lisanti et al focused on genes that were transcriptionally up-regulated by at least 1.5 fold in high compared to low density fibroblasts [70]. They performed gene-set enrichment analysis and found that high density fibroblasts demonstrated up-regulation of several cellular processes including stress signalling, stemness, angiogenesis, inflammation and fibrosis. These processes are similar to those observed in the wound healing response and could potentially mediate a pro-inflammatory and pro-fibrotic microenvironment, with high density fibroblasts sharing similar characteristics to activated myofibroblasts.

In addition, when the high density fibroblast gene signature was compared to the profile of tumour stroma, c-Jun N-terminal kinase 1 (JNK-1) stress signalling emerged as the most significant biological process shared between the two data sets [70]. The authors postulate that the stromal JNK-1 activation occurs *via* stressors in the microenvironment such as reactive oxygen species, TGF-β and fibroblast growth factor (FGF) signalling. The activated JNK-1 stress signalling mediates the onset of a ‘myofibroblast’ phenotype characterised by ongoing inflammation and fibrosis, resulting in pro-tumourigenic, high density stroma [70]. The authors also highlight a potential role for JNK-1 inhibitors as a cancer prevention strategy by inhibiting the development of the high density fibroblast phenotype.

Whether such markers of an activated stroma could be used to measure patient risk and as indicators of response to treatment strategies is an intriguing possibility.

### Mitogenesis, mutagenesis and the ‘Inactive’ stromal microenvironment gene signature

Martin and Boyd have proposed that the combined effects of cell proliferation (mitogenesis) and genetic damage to proliferating cells by mutagens (mutagenesis) may account for the increased risk of breast cancer with high MD [71]; a hypothesis similar to the concept of ‘breast tissue ageing’ proposed by Pike [17]. The Martin and Boyd model has been further adapted by Sun and colleagues, who examined stromal gene expression signatures from non-neoplastic breast tissue adjacent to invasive carcinoma in a series of breast cancer patients. They found that high MD was particularly associated with an ‘Inactive’ stromal microenvironment signature [39]. This inactive subtype was associated with increased stromal composition, higher expression of cellular adhesion genes, increased oestrogen response gene expression and reduced TGF-β signalling. Interestingly, the observation of reduced TGF-β signalling in high MD stroma is somewhat contradictory to the model proposed by Lisanti et al, discussed in the previous section. A possible explanation for this discrepancy is that the morphologically normal tissue adjacent to invasive carcinoma sampled in the study by Sun et al may be displaying aberrant gene expression, due to the proximity to the tumour. Therefore, it may not be representative of truly non-neoplastic breast tissue.

These data further emphasize the importance of stromal biology in mediating MD. The revised Martin and Boyd model incorporates and highlights the role of the stromal microenvironment in breast tumourigenesis.

Reduced TGF-β signalling in dense breast tissue has also been observed by Yang and colleagues who compared gene expression in high and low density non-tumour breast tissue taken 5cm away from invasive carcinoma [72]. A number of genes implicated in TGF-β signalling showed decreased expression in dense tissue (TGFBR2, SOS, SMAD3, CD44 and TNFRSF11B). Immunohistochemical analysis of the same tissues showed higher stromal expression of COX-2 and proliferation marker Ki67 [72]. Inhibition of TGF-β by COX has previously been reported in other organ systems [73] and loss of TGF-β ligand-mediated inhibition of mammary epithelial cell proliferation is proposed as a potential mechanism for density-associated breast cancer risk. The authors also suggest a potential role for COX-2 inhibitors as a breast cancer prevention strategy for high risk individuals. This is somewhat counter-intuitive, given the established pro-fibrogenic role of TGF-β. Furthermore, of note, the increased stromal Ki67 expression observed in this study is inconsistent with data from other groups who have reported no association of Ki67 with MD [41, 74, 75]. A recent study by Chew and colleagues has also reported increased COX-2 expression in the stromal and (to a lower degree of significance) epithelial cells of high MD breast tissue in both human samples and a xenograft model of MD [76].

**Figure 4:**
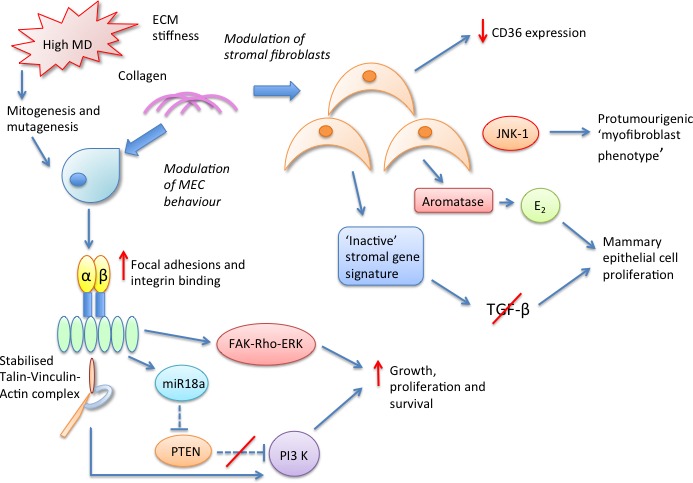
Potential stromal molecular pathways mediating the density associated cancer risk conferred by high MD breast tissue

A summary of our current understanding of the complex stromal biological pathways conferring MD-associated breast cancer risk is displayed in Figure 4 below.

Despite the progress made in these preclinical studies towards identifying potential stromal markers and signalling pathways contributing to breast density, this has not readily translated into clinically relevant markers or targets. Further clinically applicable approaches are needed to identify the key stromal molecules driving density-associated risk in humans.

## CLINICAL RELEVANCE

The influence of MD on breast cancer risk has a number of important clinical implications, particularly with regard to identification of high risk individuals, screening and prevention. Four studies have investigated whether including a measure of MD improves risk estimation compared to standard risk models [77–80]. All of these studies found a small but consistent improvement in risk estimation with the addition of MD measures, highlighting the potential clinical power of MD to accurately identify populations at increased risk of breast cancer.

Screening at more frequent intervals with more than one imaging modality may be appropriate for women with dense breasts due to their increased risk of developing cancer and their increased risk of tumour ‘masking’. A recent analysis of personalised mammographic screening according to MD, age, family history and history of breast biopsy was found to be cost effective [81].

It has been postulated that change in MD could be used as a marker in clinical trials evaluating breast cancer prevention strategies [82]. This could reduce the need for long observation periods currently required to evaluate the likelihood of developing breast cancer following an intervention. The results from a recent retrospective study by Li et al has suggested that reduction in MD can be used as a prognostic marker of response to adjuvant tamoxifen treatment [31].

MD is more strongly associated with breast cancer risk than other variables such as family history and reproductive factors. Therefore, knowledge of an individual's mammographic density and in particular, their expression levels of genes coding for ECM proteins and other stromal biomarkers, may allow individual risk prediction for breast cancer. MD also offers the advantage over other breast cancer risk factors of being modifiable, which provides exciting potential for therapeutic intervention to reduce both MD and breast cancer risk.

Targeting components of the microenvironment is already an established strategy in the adjuvant treatment setting for a number of different tumour types [83, 84]. The most advanced of these strategies are agents targeting vascular endothelial growth factor (VEGF) signalling in the tumour vasculature with a number agents now licenced for use in several metastatic cancers in combination with cytotoxic therapies [84, 85]. Beyond VEGF there are several other anti-angiogenic agents in preclinical trials targeted against platelet derived growth factor (PDGF) and FGF signalling pathways [86, 87].

Components of tumour-associated inflammation are also a focus of new agents targeted against the microenvironment. An interleukin 6 (IL-6) neutralising antibody is currently undergoing evaluation in clinical trials [88], and neutralising antibodies against human chemokines have shown promising results in preclinical models of prostate and breast cancer [89]. Disrupting tumour-stroma crosstalk *via* integrin inhibition is also the subject of clinical trials with agents showing limited anti-tumour efficacy at present [90, 91]. Specifically targeting tumour-associated fibroblasts, utilising their expression of fibroblast activation protein α (FAP), remains at the preclinical stage of development. Such strategies involve; the development of anti-FAP antibodies conjugated to cytotoxic drugs [92], utilising the enzymatic activity of FAP to activate pro-drugs in the vicinity of the tumour [93], and vaccines targeted against FAP [94].

Harnessing similar approaches in the preventive setting to target the stromal molecules and pathways mediating MD could provide an effective cancer prevention strategy. Thus, further work to dissect the complex biological pathways mediating MD is urgently needed to identify novel, clinically relevant, biomarkers driving the density-associated risk.
